# Enolase-1 is a therapeutic target in endometrial carcinoma

**DOI:** 10.18632/oncotarget.3639

**Published:** 2015-04-24

**Authors:** Mengyang Zhao, Weiyi Fang, Yan Wang, Suiqun Guo, Luyun Shu, Lijing Wang, YiYu Chen, Qiaofen Fu, Yan Liu, Shengni Hua, Yue Fan, Yiyi Liu, Xiaojie Deng, Rongcheng Luo, Zhong Mei, Qinping Jiang, Zhen Liu

**Affiliations:** ^1^ Cancer Center, Traditional Chinese Medicine-Integrated Hospital of Southern Medical University, Guangzhou, Guangdong, China; ^2^ Cancer Research Institute, Southern Medical University, Guangzhou, China; ^3^ Department of Pathology of Basic School, Medical University of Guangzhou, Guangzhou, China; ^4^ Department of Pathology, Third Affiliated Hospital of Guangzhou Medical College, Guangzhou, China; ^5^ Department of Obstetrics and Gynecology of Nanfang Hospital, Southern Medical University, Guangzhou, China; ^6^ Department of Obstetrics and Gynecology, the Third Affiliated Hospital of Southern Medical University, Guangzhou, China

**Keywords:** ENO1, endometrial carcinoma, cell growth, EMT, glycolysis

## Abstract

ENO1 plays a paradoxical role in driving the pathogenesis of tumors. However, the clinical significance of ENO1 expression remains unclear and its function and modulatory mechanisms have never been reported in endometrial carcinoma (EC). In this study, ENO1 silencing significantly reduced cell glycolysis, proliferation, migration, and invasion *in vitro*, as well as tumorigenesis and metastasis *in vivo* by modulating p85 suppression. This in turn mediated inactivation of PI3K/AKT signaling and its downstream signals including glycolysis, cell cycle progression, and epithelial-mesenchymal transition (EMT)-associated genes. These effects on glycolysis and cell growth were not observed after ENO1 suppression in normal human endometrial epithelial cells (HEEC). Knocking down ENO1 could significantly enhance the sensitivity of EC cells to cisplatin (DDP) and markedly inhibited the growth of EC xenografts *in vivo*. In clinical samples, EC tissues exhibited higher expression levels of ENO1 mRNA and protein compared with normal endometrium tissues. Patients with higher ENO1 expression had a markedly shorter overall survival than patients with low ENO1 expression. We conclude that ENO1 favors carcinogenesis, representing a potential target for gene-based therapy.

## INTRODUCTION

Endometrial Carcinoma (EC) is the most common gynecological malignancy and the fourth most common malignancy in women in the developed world today [1]. EC is often associated with excessive estrogen exposure, and often coexists with, or is preceded by endometrial hyperplasia [2]. In China, the incidence of EC has been increasing with a shift towards younger populations due to multiple factors, such as obesity and lifestyle changes [3, 4]. Upon initial diagnosis, approximately 28% of patients already have regional or distant metastasis [5]. The transition from normal endometrium to carcinoma is thought to occur through a progression of alterations in genes involving cell proliferation, inhibition of apoptosis, and angiogenesis. Therefore, further elucidation of the molecular mechanisms during endometrial cancer is urgently required.

Enolase, also known as pyruvate dehydrogenase phosphatase, catalyzes not only the transformation of 2-phosphate-d-glycerate to phosphoric acid-pyruvate during glycolysis but also the reverse conversion of phosphoric acid-pyruvate to 2-phosphate-d-glycerate during glycogen synthesis [6]. Thus, enolase plays a critical role in anaerobic glycolysis. The alpha-enolase (ENO1), as one of three enolase subunits that plays a functional role in several physiological processes depending on its cellular localization [7] and intrinsically encodes both α-enolase and a shorter monomeric structural lens protein, tau-crystallin. α-enolase is a full-length form that is found in the cytoplasm. The shorter tau-crystallin protein is produced from an alternative translation start of ENO1, and it is localized to the nucleus. This protein has been found to bind to an element in the c-Myc promoter [8], and has been designated as the c-Myc promoter-binding protein (MBP-1). In a previous study, ENO1 was upregulated and activated by several glucosetransporters and glycolyticenzymes that contribute to the Warburg effect in tumor cells [9]. Increased ENO1 gene activity and protein production has been detected in several tumors [10–14]. However, the role of ENO1 in EC remains unknown.

In order to further validate the role of ENO1 and its molecular basis in EC, we evaluated the expression of ENO1 in human normal endometrium (NE) and EC tissues, as well as its effects on cell glycolysis, growth, migration, and invasion *in vitro* as well as tumorigenicity and metastasis *in vivo*. Our studies showed that ENO1 is overexpressed in EC tissues, which was markedly unfavorable for the prognosis of EC patients. Further, downregulated ENO1 inhibited cell glycolysis, proliferation, migration, invasion, and tumorigenicity via inactivation of PI3K/AKT pathway mediated by p85 suppression. Furthermore, suppression of ENO1 could significantly enhance the sensitivity of EC cells to cisplatin (DDP) *in vitro* and *in vivo*. Our findings demonstrate that ENO1 acts as a potential oncogene and a novel therapeutic target in EC.

## RESULTS

### Stablely knocking down ENO1 expression inhibits glycolysis and cell proliferation in EC cells but not HEEC

To gain an insight into the role of ENO1 in EC, we first used a lentiviral shRNA vector to specifically and stably knock down the expression of ENO1 in two EC cells (HEC-1B and Ishikawa) (Supplementary Figure 1A). Transcriptional levels of ENO1 were assessed by RT-PCR, with efficient knockdowns from shENO1-A in HEC-1B cell line and shENO1-C in Ishikawa cell line compared to the empty vector controls [pLVTHM-GFP-Control (PLVTHM)] (*P* < 0.05) (Supplementary Figure 1B). Consistent results for protein level decreases were observed by western blotting (Supplementary Figure 1C). Protein levels of the HEECs treated with siENO1 and negative control (NC) were also analyzed (Supplementary Figure 1D).

**Figure 1:**
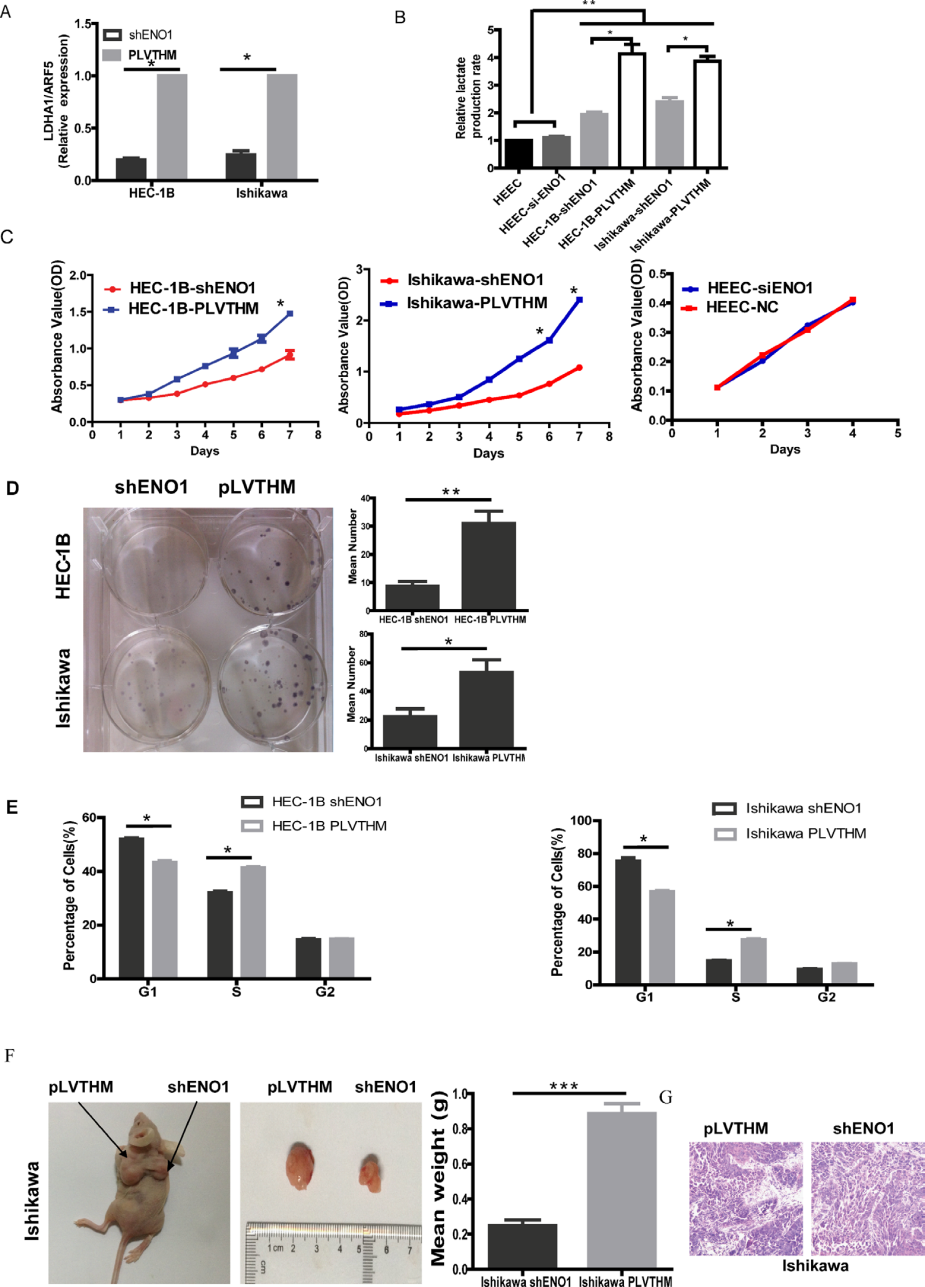
Knocking down ENO1 expression suppressed glycolysis and cell proliferation in EC cells but not HEEC **A.** RT-PCR showing transcriptional level of the LDHA with ARF5 used as a loading control. Bar graph showed the relative expression of mRNA among the groups. Data were presented as mean ± SEM for three independent experiments (*P < 0.05). **B.** The levels of lactate production were examined in HEEC, EC cell lines and ENO1 sliencing EC cells using the Automatic Biochemical Analyzer (#7170A, HITACHI, Japan). Data were shown as mean ± SEM of three experiments(*P < 0.05, **P < 0.01). **C.** Effect of ENO1 knockdown on EC cells or HEEC cell proliferation as measured by MTT assay. Absorbance was read at 490 nm with averages shown from triplicate wells. Data were presented as mean ± SEM for three independent experiments (*P < 0.05). **D.** *In vitro* proliferative ability of EC cells was significantly decreased in ENO1-suppressed cells compared to PLVTHM cells by colony formation assay (*P < 0.05, **P < 0.01). **E.** ENO1-suppressed dramatically reduced cell cycle progression from G1 to S phase. Data were presented as mean ± SEM for three independent experiments (*P < 0.05). **F.** When compared with PLVTHM, *in vivo* tumorigenicity of shENO1-Ishikawa cells was markedly reduced (*P < 0.05). **G.** HE staining in subcutaneous tumors of mice injected with shENO1 or PLVTHM cells.

To assess the metabolic changes triggered by ENO1, we used QPCR to detect the expression of lactate dehydrogenase A (LDHA) in EC cells and HEECs after ENO1 knockdown. The mRNA level of LDHA was significantly decreased in sh-ENO1 EC cells (Figure 1A). To further confirm our data, we examined the level of lactate production in EC and HEEC cell lines. Consistent with the QPCR results, sh-ENO1-HEC-1B and Ishikawa cells extruded a smaller amount of lactate (Figure 1B) into the media compared to control cells. The level of LDHA was not affected in HEEC-siENO1, suggesting that normal cellular processes contributing to proton production in the media independent of glucose metabolism are not influenced by the absence of ENO1.

Subsequently, we examined the effect of decreased ENO1 expression on EC cells and HEEC growth *in vitro*. Using an MTT assay, we found that the growth of shENO1 HEC-1B and Ishikawa cells was significantly slower than the PLVTHM cells from day 4 (*P* < 0.05), but the growth of HEEC was not influenced by absence of ENO1 (Figure 1C). Further, we also found that elevated ENO1 induced cell proliferation in HEEC (Supplementary Figure 2A and 2B).

Colony formation assays showed that suppressing ENO1 significantly decreased cell proliferation compared to PLVTHM cells (Figure 1D). Cell cycle analysis showed that ENO1 suppression dramatically reduced cell cycle progression from G1 to S phase (Figure 1E). To confirm the growth enhancing effects of ENO1, we performed an *in vivo* tumorigenesis study by inoculating shENO1 Ishikawa cells into nude mice. Mice in the shENO1-Ishikawa and PLVTHM groups were sacrificed 20 days after inoculation, with average tumor weights of 0.247g and 0.887g, respectively (*P* < 0.001) (Figure 1F). HE staining of subcutaneous tumors from shENO1 and PLVTHM injected cells is shown in Figure 1G.

### Stable knockdown of ENO1 attenuates cell migration and invasion *in vitro* and *in vivo* in EC cells

**Figure 2:**
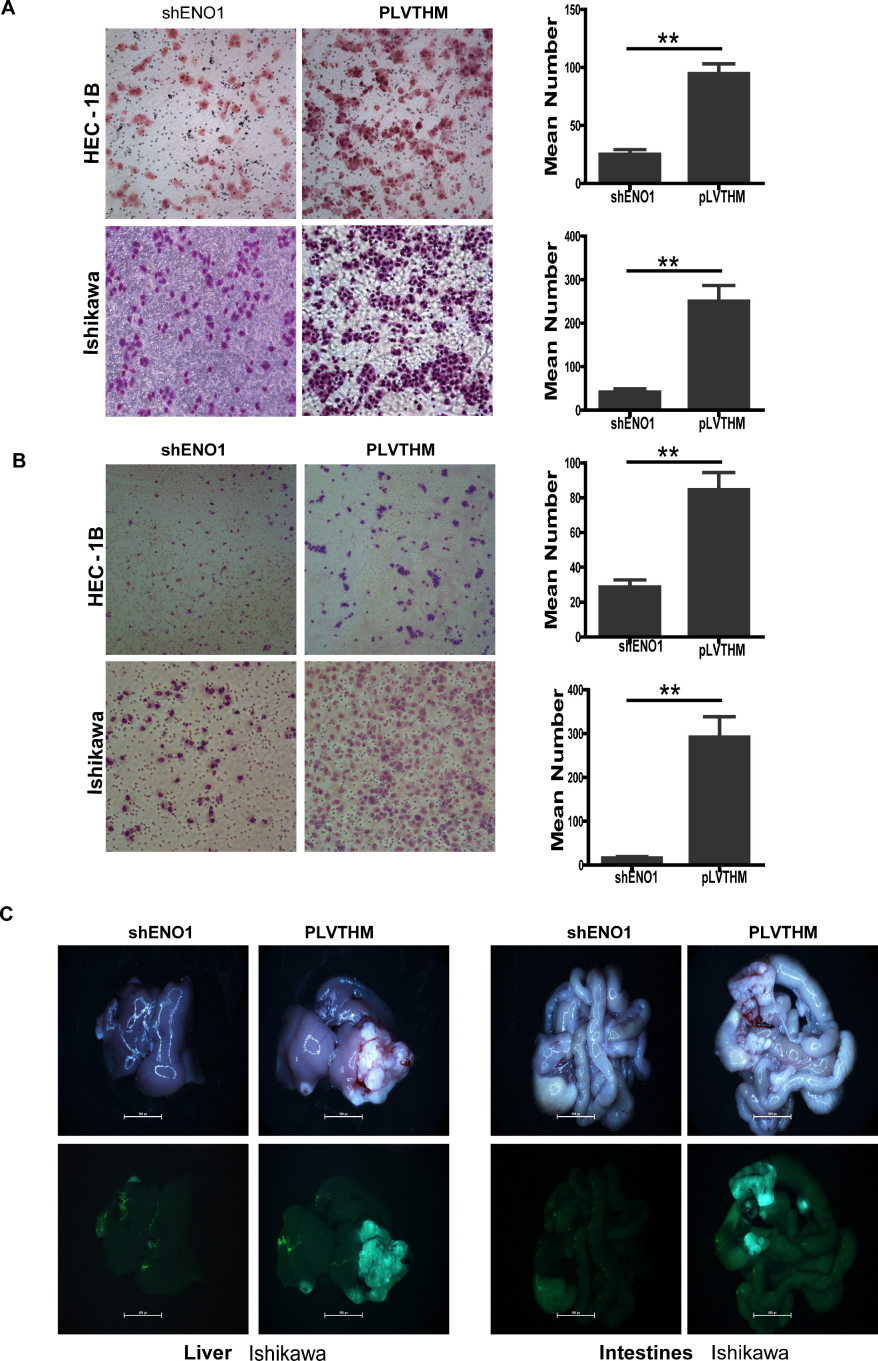
Stablely knockdown of ENO1 attenuated cell migration and invasion *in vitro* and *in vivo* in EC **A.** Stably downregulating ENO1 reduced the migration ability of EC cells *in vitro* (**P < 0.01). **B.** Stably suppressed ENO1 reduced *in vitro* invasiveness of EC cells. Data were presented as mean ± SEM for three independent experiments. **P < 0.01 signified a statistically significant difference. **C.** External whole-body fluorescence images of liver and intestines were obtained 2 months after spleen injection, respectively.

To examine the effect of ENO1 on cell migration, shRNA-ENO1 infected HEC-1B and Ishikawa EC cells were cultured on a Transwell apparatus. After 15 h incubation, the percentage of migrated cells in both shENO1-HEC-1B and shENO1-Ishikawa EC cell groups was significantly less than the PLVTHM groups (for both *P* < 0.01) (Figure 2A). Using a Boyden chamber coated with matrigel, we determined changes in cell invasiveness after 16 h incubation. Compared with the PLVTHM cells, shRNA-ENO1 HEC-1B and Ishikawa EC cells both showed significantly decreased invasiveness (*P* < 0.01 for each) (Figure 2B).

To assess the effect of ENO1 on EC metastasis *in vivo*, ENO1-depleted Ishikawa cell and the control cells were independently injected into the spleens of nude mice. Fluorescence images showed a few scattered metastatic nodules in livers and intestines of nude mice were formed in the mice. The results showed extensively distributed large metastatic nodules in mice injected with PLVTHM cells, while only a few scattered metastatic cells were observed on the section of the mice injected with shENO1 cells (Figure 2C).

### Transient knockdown of ENO1 with siRNAs inhibits cell proliferation, migration and invasion *in vitro* in EC cells

**Figure 3:**
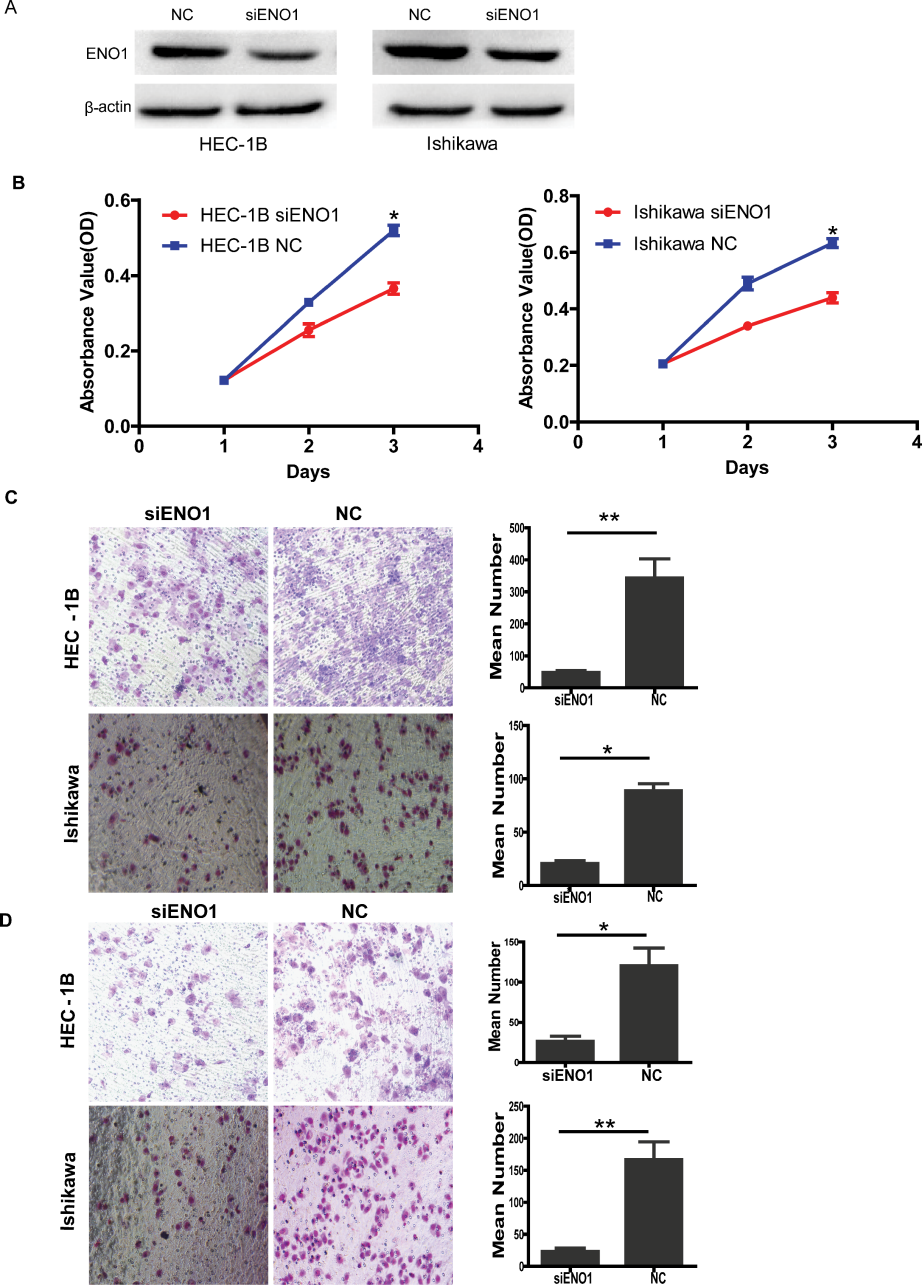
Transiently reducing the expression of ENO1 by siRNA inhibited cell proliferation, migration and invasion **A.** Efficiency of siRNA knock down of ENO1 in EC cell lines. **B.** Transiently reducing the expression of ENO1 by siRNA inhibited cell proliferation in EC cells. **C.** Transiently downregulated ENO1 dramatically decreased the migration ability of EC cells *in vitro*. **D.** Transiently suppressed ENO1 inhibited *in vitro* invasiveness of EC cells. Data were presented as mean ± SEM for three independent experiments (*P < 0.05, **P < 0.01).

Interestingly, similar results were also observed in siRNA-mediated suppression of ENO1 in EC cells. The protein levels of ENO1 were assessed by western blotting (Figure 3A). We found that knocking down endogenous ENO1 expression decreased cell proliferation compared to the NC groups (for both *P* < 0.05) (Figure 3B). siRNA-mediated suppression of ENO1 also suppressed cell migration and invasion compared to the NC groups (*P* < 0.05 and *P* < 0.01, respectively) (Figure 3C and D).

### ENO1 regulates the expression of glycolysis, cell cycle and EMT-associated genes in EC cells

**Figure 4:**
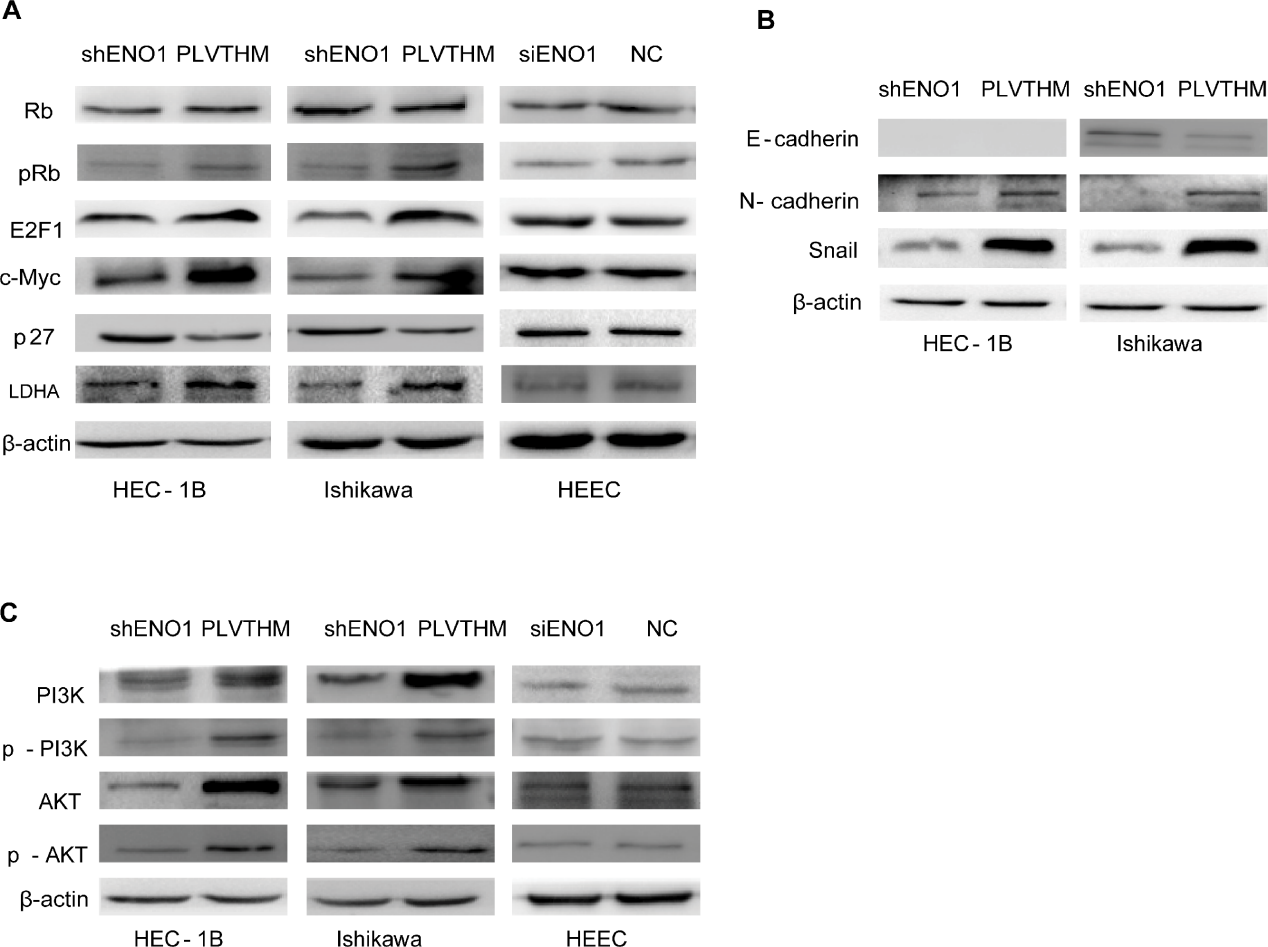
ENO1 controlled the expression of glycolysis, cell cycle and EMT-associated genes in EC via the PI3K/AKT pathway but not HEEC **A.** Knocking down endogenous ENO1 expression reduced the expression of c-Myc, E2F1, pRb (Ser780) and LDHA, and enhanced p27 in EC cells. However, total Rb was not affected. The expression of Rb, c-Myc, E2F1, pRb (Ser780), p27 and LDHA were not influenced in ENO1 silencing HEEC. **B.** Suppressing ENO1 expression decreased the expression of EMT marker genes Snail and N-cadherin and enhanced E-cadherin expression. Protein level of E-cadherin was not examined in the HEC-1B cell line. **C.** Reduced ENO1 expression decreased the expression of pPI3K, and pAKT, as well as p85 and AKT protein levels. The levels of PI3K, AKT, p-PI3K and p-AKT in ENO1 silencing HEEC were not affected. β-actin served as the internal control. Each experiment was repeated three times.

To further study the mechanism by which ENO1 regulates cell glycolysis, proliferation, migration, invasion, and metastasis, we assessed relevant factors after ENO1 silencing in EC cells. We observed that the protein level of cell glycolysis-associated LDHA was significantly decreased in sh-ENO1 EC cells as well as cell-cycle regulators c-Myc, p-Rb, and E2F1. Conversely, cell-cycle inhibitor P27 was highly expressed after stable ENO1 suppression (Figure 4A). Surprisingly, the expression levels of LDHA, p-Rb, c-Myc, E2F1, and P27 did not display any significantly changes in ENO1-depleted HEECs compared to control cells (Figure 4A). Further, we found that mesenchymal markers Snail and N-cadherin were weakly expressed and epithelial marker E-cadherin was highly expressed in the EC cells upon reduced expression of ENO1 (Figure 4B). However, stably downregulated ENO1 did not induce any epithelial to mesenchymal transition morphology changes in Ishikawa nor HEC-1B cells (Supplementary Figure 1A).

### ENO1 regulates P85α-mediated PI3K/AKT pathway to promote cell glycolysis, proliferation, migration, and invasion

PI3K/Akt constitutes an important pathway modulating multiple biological processes [15]. To address the mechanism of ENO1-mediated phenotypic changes, we examined its effect on the PI3K/AKT pathway by western blotting. We found that reduced ENO1 significantly decreased the expression of phosphorylated PI3K p85 (Tyr458) and AKT (Ser473), and also decreased PI3K (p85α) and AKT protein levels (Figure 4C). There were not any changes in PI3K, AKT, p-p85 or p-AKT in siENO1-HEEC (Figure 4C).

**Figure 5:**
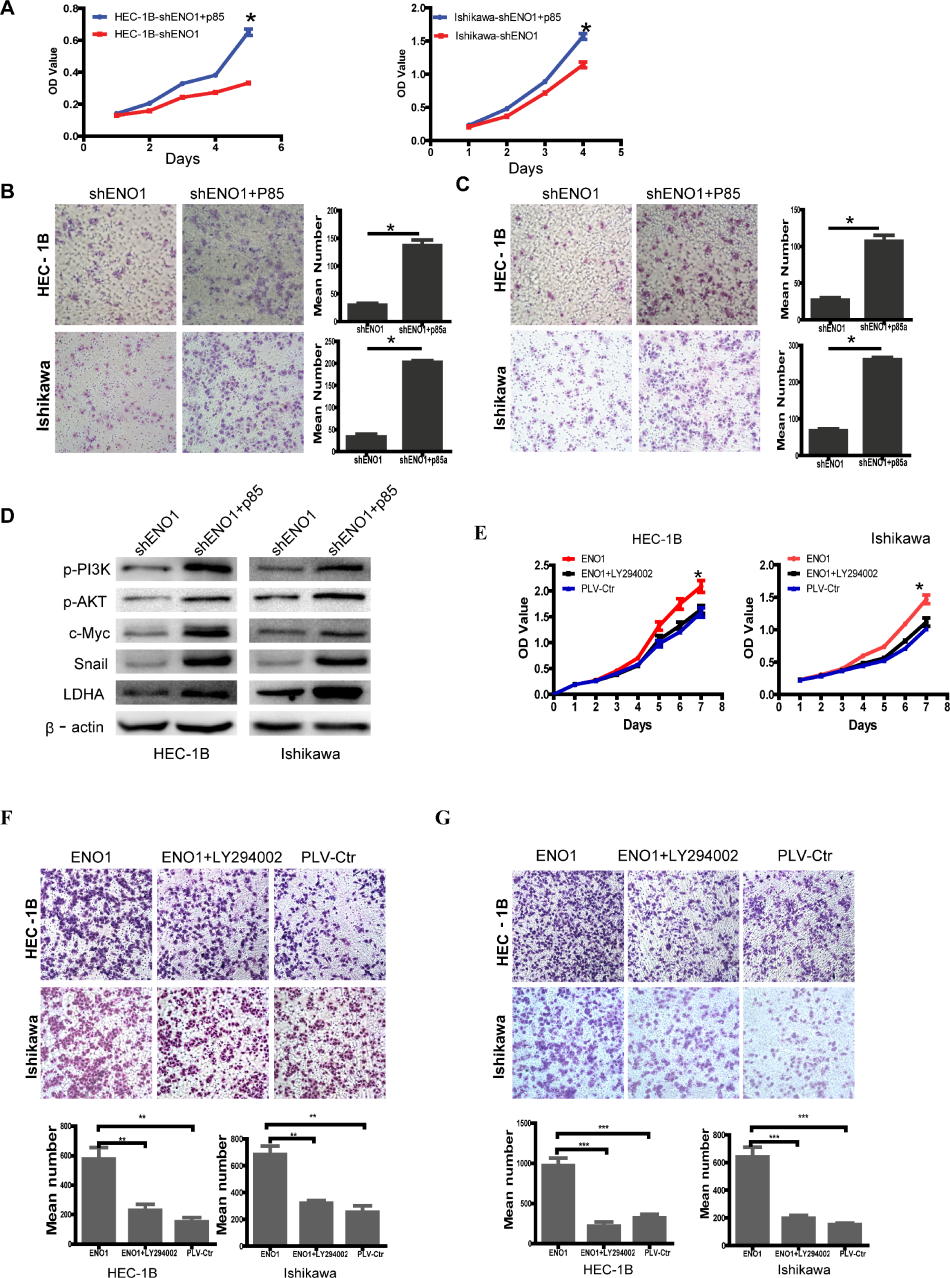
Overexpressed PI3K with p85α plasmid reverses the effect of stably downregulated ENO1 and suppressed p-PI3K with Ly294002 (10 μM) reverses the effect of stably upregulated ENO1 in EC cells **A–C.** Overexpressed p85α in ENO1-suppressed EC cells restored cell proliferation, migration and invasion compared with ENO1 sliencing EC cells. **D.** Overexpressed p85α in ENO1-suppressed EC cells induced the protein levels of p-PI3K, p-AKT, c-Myc, Snail and LDHA. **E–G.** The promoting effect of stably overexpressed ENO1 in cell proliferation, migration and invasion was reversed by Ly294002 (10 μM) treatment. Data were presented as mean ± SEM for three independent experiments (*P < 0.05, **P < 0.01, ***P < 0.001).

To study the mechanism by which ENO1 regulates cell glycolysis, proliferation, migration, invasion and metastasis, ENO1-suppressed EC cells were treated with p85 plasmid to induce the phosphorylation of PI3K (Supplementary Figure 3A). Overexpressed PI3K by p85α plasmid in ENO1-suppressed EC cells enhanced cell glycolysis, proliferation, migration, invasion and metastasis by increasing the expression of p-PI3K, p-AKT, c-Myc, Snail and LDHA compared to ENO1 silenced EC cells (Figure 5A–5D). Plasmid with p85α cDNA was also transfected into HEEC cells which stimulated their growth (Supplementary Figure 3B and C). Subsequently, we found that the positive effect of stable ENO1overexpression on cell proliferation, migration and invasion was reversed by Ly294002 (10 μM) treatment (Figure 5E–5G). These results suggested that ENO1 acts as an upstream factor which promotes cell glycolysis, proliferation, migration, invasion, and metastasis by modulating the PI3K/AKT via p85 in EC.

**Figure 6:**
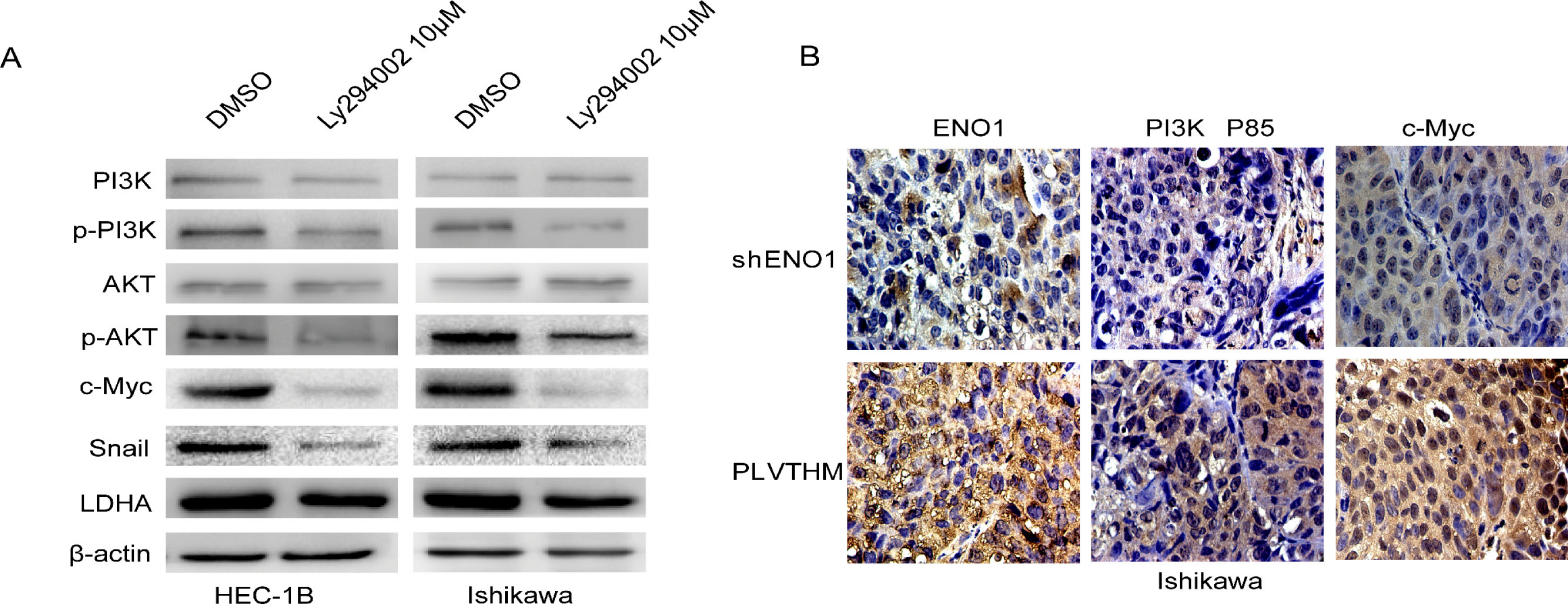
ENO1 regulates PI3K/AKT pathway and its downstream signal moleculars in EC **A.** Suppressing the expression of PI3K by its specific inhibitor Ly294002 (10 μM) reduced pPI3K, pAKT, c-Myc, Snail and LDHA expression in EC cells, but did not induce PI3K, AKT expression change. β-actin served as the internal control. Each experiment was repeated three times. **B.** The expressions of ENO1, PI3K(P85) and c-Myc were evaluated by immunohistochemistry in tumor tissues derived from the mouse models of sh-ENO1 Ishikawa and the control models. Magnification, × 400.

In addition, we used specific inhibitor Ly294002 (10 μM) to suppress the expression of phosphorylated PI3K and observed decreased expression of c-Myc, Snail and LDHA in EC cells (Figure 6A). Further, the expression of ENO1, PI3K(P85α) and c-Myc were evaluated by immunohistochemistry in xenografts from EC and control models. Notably, in three weeks after implantation, tumors from mice injected with sh-ENO1 Ishikawa cells appeared to display relatively lower expression levels of PI3K (P85α) and c-Myc in tumor tissues relative to the controls (Figure 6B).

### Silencing of ENO1 enhances DDP chemosensitivity to EC cells *in vitro* and *in vivo*

**Figure 7:**
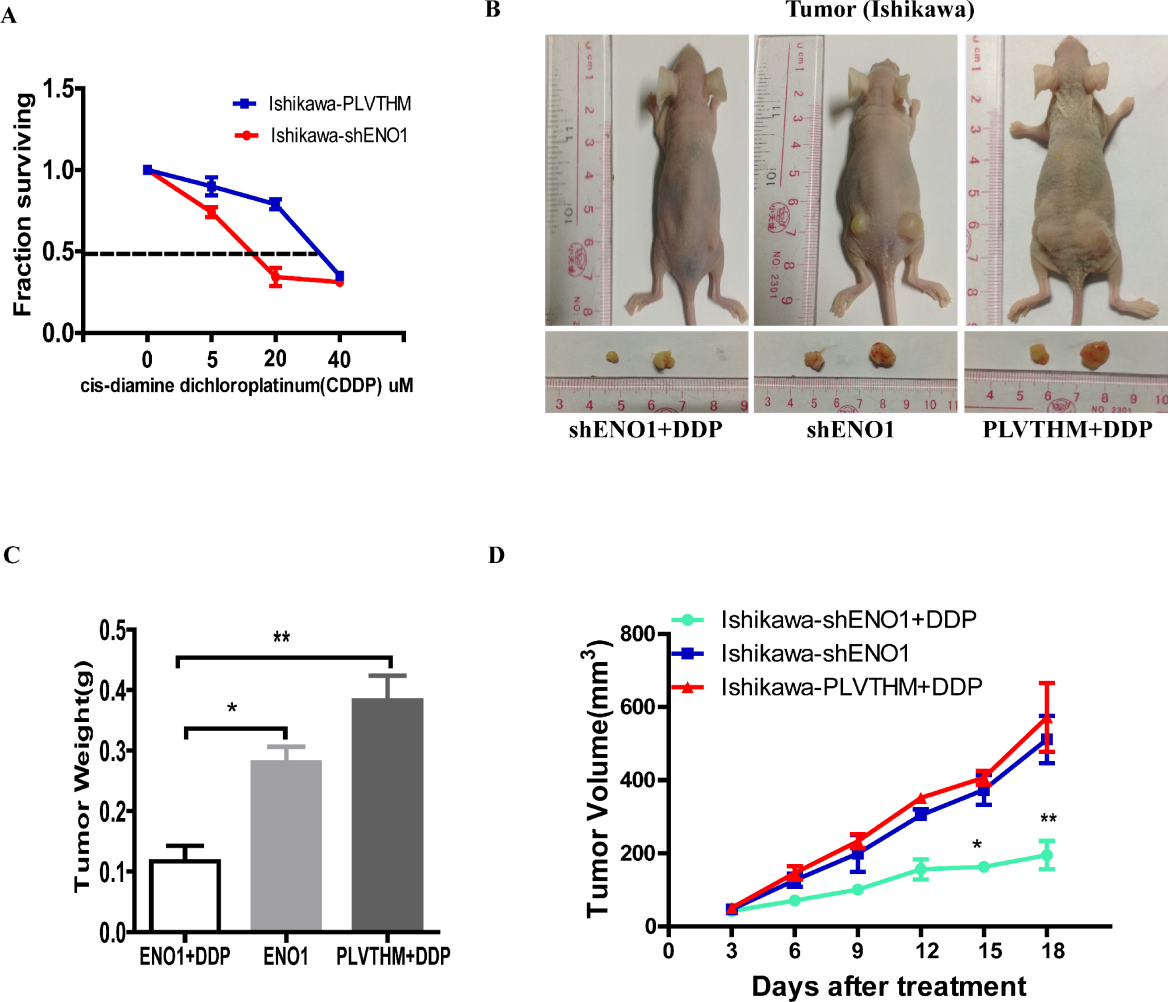
Silencing of ENO1 enhances DDP chemosensitivity *in vitro* and *in vivo* **A.** Dose-response curves of Ishikawa treated by shENO1 and PLVTHM respectively following 48 h treatment with DDP. **B, C.** The *in vivo* effectiveness of DDP was evaluated in xenograft mouse models bearing tumors originating from Ishikawa-shENO1 and Ishikawa-PLVTHM cells. **D.** The tumor volume was periodically tested for each mouse and tumor growth curve was plotted. Data were shown as the mean ± SEM (*P < 0.05, **P < 0.01).

The EC cell line exhibited significantly increased sensitivity to DDP after stable knockdown of ENO1. Growth inhibition rates induced by different concentrations of DDP after 48 h treatment were calculated before and after ENO1 silencing, and a fitted curve was obtained for the determination of IC50. These results indicated that the IC50 of DDP was 37.83 μM in the parental Ishikawa cells while reduced to 17.1 μM in ENO1 silencing EC cells (Figure 7A).

Further, we evaluated the *in vivo* efficacy of DDP in mice bearing tumors originating from shENO1-Ishikawa cells and PLVTHM-Ishikawa cells. Consistently, we observed that ENO1-silenced groups treated with DDP exhibited larger tumor growth inhibition than the control groups treated with DDP (Figure 7B–7D).

### Expression of ENO1 gene in EC tissues and NE tissues

**Figure 8:**
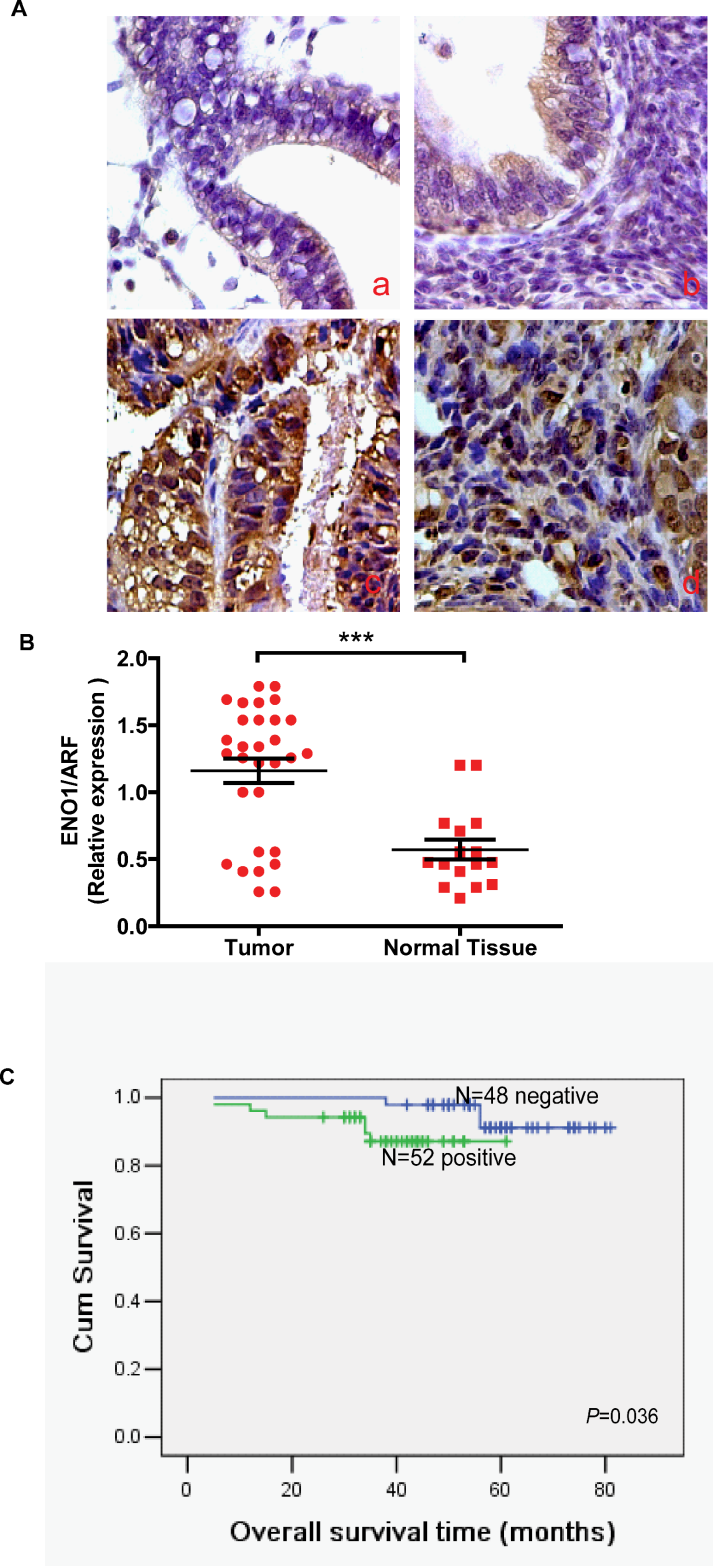
ENO1 protein was expressed in the cytoplasm of NE, endometrial atypical hyperplasia (EAH) and EC samples (original magnification: 400X) **A.** a). Negative staining of ENO1 in NE. b). Weak staining of ENO1 in EAH. c-d). Strong cytoplasmic ENO1 staining in EC. **B.** mRNA expression of ENO1 was decreased in NE tissues compared with EC tissues by real-time PCR. Data were presented as mean ± SEM for three independent experiments (***P < 0.001). **C.** Expression of ENO1 protein predicts EC patients' overall survival. Patients with positive ENO1 had worse survival than those with negative ENO1 (*P* = 0.036).

In order to further assess the role of ENO1 in EC, we measured the expression levels and subcellular localization of ENO1 protein in 20 normal NE tissues, 22 endometrial atypical hyperplasia (EAH) and 100 archived paraffin-embedded EC samples using immunohistochemical staining (Figure 8A). We found that ENO1 was weakly expressed in cytoplasm in NE tissues compared to EC tissues. ENO1 protein was highly expressed in 52% (52/100) of EC samples and 31.8% (7/22) of EAH samples, while only in 15.0% (3/20) of NE samples, a significantly lower frequency (*P* = 0.005) (Table 1). We also performed real-time PCR to measure the expression of ENO1 mRNA transcripts in 30 freshly collected EC tissues and 16 freshly collected NE tissues. Compared with NE tissues, EC tissues exhibited higher expression levels of ENO1 mRNA (*P* < 0.001) (Figure 8B).

**Table 1:** Protein expression of ENO1 in NE, EAH and EC samples

Group	Cases	Protein expression		*P* value
		Low expression	High expression	
NE	20	17	3	
EAH	22	15	7	
EC	100	48	52	0.005

### Relationship between clinicopathologic characteristics and ENO1 expression in EC patients

The relationship between clinicopathologic characteristics and ENO1 expression levels in individuals with EC is summarized in Table 2 and Table 3. We did not find a significant association between ENO1 expression levels and patients' age, sex, histologic type, FIGO stage in the 100 EC cases. However, we observed that the expression level of ENO1 was positively correlated with lymph node status (*P* = 0.005) (Table 2) and depth of myometrial invasion (*P* = 0.033) (Table 3) in EC patients.

**Table 2:** Correlation between the clinicopathologic characteristics and expression of ENO1 protein in EC

ENO1(%)				
Characteristics	N	Lowexpression	High expression	*P* value
Age				
<50	34	15(44.1%)	19(55.9%)	
≧50	66	33(50%)	33(50%)	0.579
Family history of tumor				
Negative	85	41(48.2%)	44(51.3%)	
Positive	15	4(26.7%)	11(73.3%)	0.222
Menopausal status				
Premenopausal	53	29(54.7%)	24 (45.3%)	
Postmenopausal	47	19(40.4%)	28(59.6%)	0.156
Complications				
Without	70	34(48.6%)	36(51.4%)	
With	30	14(46.7%)	16(53.3%)	0.863
FIGO stage				
I–II	77	40(51.9%)	37(48.1%)	
III	23	8(34.8%)	15(65.2%)	0.150
Depth of myometrial invasion				
<50%	66	35(53.0%)	31(47.0%)	
≧50%	34	13(38.2%)	21(61.8%)	0.163
Lymph node status				
Negative	80	44(55.0%)	36(45.0%)	
Positive	20	4(20.0%)	16(80.0%)	0.005

**Table 3:** Summary of univariate and multivariate Cox regression analysis of overall survival duration

Parameter	Univariate analysis			Multivariate analysis		
	P	HR	95%CI	P	HR	95%CI
Age						
<50 vs. ≧50	0.879	1.114	0.278–4.463			
Family history of tumor						
Negative vs. Positive	0.443	0.039	0.001-149.16			
Menopausal status						
Premenopausal vs. Postmenopausal	0.64	0.715	0.176-2.905			
complications						
with vs. without	0.215	0.268	0.033-2.149			
FIGO stage						
I+II vs. III	0.027	4.407	1.182-16.434	0.537	0.507	0.059-4.381
Lymph node status						
Negative vs. Positive	0.004	7.019	1.856-26.534	0.105	6.806	0.67-69.175
Depth of myometrial invasion						
<50% vs. ≧50%	0.014	7.107	1.476-34.217	0.033	5.668	1.148-27.987
ENO1 expression						
Low vs. high	0.067	4.982	0.893-27.789	0.551	1.727	0.287-10.377
Postoperative irradiation						
Yes vs.No	0.997	0.996	0.122-8.155			
Postoperative chemotherapy						
Yes vs.No	0.041	3.958	1.06-14.781	0.185	2.568	0.636-10.358
Postoperative hormone therapy						
Yes vs.No	0.126	0.293	0.061-1.415			

### ENO1 high expression is associated with overall survival of EC

To investigate the prognostic value of ENO1 expression in EC, we assessed the association between levels of tumor ENO1 expression and patients' survival using Kaplan-Meier analysis with the log-rank test. In 100 EC cases with prognosis information, we observed that the level of ENO1 protein expression was significantly correlated with overall survival. Patients with high expression had worse prognoses than those with low expression of ENO1 (Figure 8C) (*P* = 0.036).

## DISCUSSION

It is widely accepted that increased glucose uptake and aerobic glycolysis are features of many tumors and that targeting key metabolic enzymes of these pathways poses a therapeutic opportunity. For cancer cells to sustain their rapid proliferation and gain a survival advantage, glycolysis has been demonstrated as a hot spot for infection-mediated metabolic reprogramming [16–20]. The ENO1 gene, as one of the prominent enzymes of the glycolytic pathway, catalyzes the conversion of 2-phosphoglycerate into phosphoenolpyruvate [21] and binds to several glycolytic enzymes including pyruvate kinase, phosphoglycerate mutase, aldolase and microtubule network proteins to potentially modulate multitude of functions [22]. Interestingly, ENO1 plays different roles in different types of cancer. In hepatocellular carcinoma, head and neck cancer, gynecological malignancies, ENO1 has been shown to be an oncogenic factor promoting tumor progression [23–25]. Conversely, ENO1 functions as a tumor suppressor in NSCLC [26]. However, the role of ENO1 in EC remains unknown.

In order to evaluate the function of ENO1 and eliminate the influence of MBP-1 on EC, we firstly performed an immunofluorescence and observed that ENO1 was expressed in the cytoplasm but not in the nucleus (MBP-1) in Ishikawa and HEC-1B cells. Furthermore, we also found that MBP-1 was not expressed by Western blotting assay in Ishikawa, HEC-1B and HEEC cells. The above mentioned results suggested that the three cells could be used as well-defined models to evaluate the function of ENO1. Further, stable ENO1-suppressed Ishikawa and HEC-1B cells as well as transient ENO1-suppressed HEEC cells were respectively constructed, which was used to investigate the role of ENO1 in EC and HEEC.

ENO1 was originally described as an enzyme responsible for the glycolytic pathway. To further assess the effect of ENO1 on EC cells and HEEC, we analyzed the glycolysis changes triggered by ENO1 and found that suppressed ENO1 decreased the production of lactate in EC. Interestingly, the significant change of extrusion of lactate was not observed in siENO1-HEEC. These data suggested that ENO1 was involved in inducing glycolysis in EC but not HEEC. In addition, we found that decreased expression of ENO1 reduced the proliferation of EC cells but not HEEC. Further, we also found that elevated ENO1 induced cell proliferation in HEEC. These above-mentioned results suggested that increased ENO1 functioned as a potential oncogene promoting tumor growth by inducing glycolysis in EC, but not in HEEC with its normal physiology expression condition. Further, we discovered that knocking down ENO1 inhibited cell migration, invasion, and metastasis in EC. However, we did not observe any EC cell morphology changes, a result consistent with our previous report of ENO1 function in glioma [27].

The biological functions of ENO1 found in this study provided a basis for the mechanistic observations. c-Myc has been shown to regulate LDHA at the transcriptional level [28] and could reprogram the core energy metabolism by regulating LDHA, leading to greater nutrient uptake and increased macromolecular biosynthesis [29–31]. Furthermore, c-Myc can induce the expression of p-RB and stimulate its interaction with E2F1 [32] by inhibiting P27^Kip1^ [33–35], which accelerates cell cycle transition from G1 to S phase [36] and thus promotes cell proliferation. Snail is a key transcriptional factor that represses the expression of epithelial marker gene E-cadherin by binding to the E-Box site in its promoter [37] and induces the expression of mesenchymal marker N-cadherin [38] which stimulates cell migration, invasion, and metastasis in tumors. Interestingly, c-Myc and Snail have been reported as the key downstream activation genes of PI3K/AKT pathway and induce glycolysis, proliferation, migration, invasion and metastasis in tumors [39–42]. In this study, we firstly observed that ENO1 silencing significantly decreased the expression of c-Myc, p-Rb, E2F1, LDHA, Snail, and N-cadherin but upregulated P27^Kip1^ and E-cadherin expression (E-cadherin was not examined in HEC-1B cell line). Furthermore, we observed that decreased ENO1 expression suppressed p-PI3K and p-AKT protein levels in EC, which was consistent with our findings in glioma [27]. Inhibition of PI3K/AKT signaling significantly suppressed the protein expression of c-Myc and its downstream gene LDHA as well as Snail in EC cells. Interestingly, the protein levels of P85α and AKT were also found to be reduced in ENO1-suppressed EC cells which we did not observe before. We hypothesizes that ENO1 plays a potential carcinogenic role through the P85α-mediated activation of PI3K/AKT pathway in EC. To clarify hypothesis, the plasmid with p85α cDNA was firstly transfected into HEEC cells and stimulated its growth. Further, overexpressed p85α in ENO1-suppressed EC cells not only restored cell malignant phenotypes, but also induced the protein levels of p-PI3K and p-AKT. Furthermore, specific inhibitor of pPI3K Ly294002 was transfected into ENO1-overexpressed cells and markedly blocked ENO1-mediated cell malignant phenotypes. Taken together, the above-mentioned results demonstrated that ENO1 acted as a potential oncogene by activating p85-mediated PI3K/AKT and its downstream glycolysis, cell cycle, and EMT signals in EC.

As one of the most widely used chemotherapy dugs for EC among those containing platinum drugs, DDP is believed to induce tumor cell death resulting from the formation of DDP-DNA adduts, which inhibit DNA replication and transcription [43]. However, the intrinsic or acquired resistance to DDP in EC cells remains a major obstacle to successful chemotherapy. It is defined that the mechanism of resistance to DDP is multifactorial, many genes or gene products have been affirmed to the responsible for DDP resistance [44]. In this study, we verified that ENO1 silencing could enhance the sensitivity of DDP *in vitro* and *in vivo*, suggesting that the therapeutic effect of DDP was significantly better in EC patients with lower expression of ENO1.

In previous studies, elevated expression of ENO1 was positively associated with progression and poor prognosis in neuroendocrine tumors, neuroblastoma, pancreatic cancer, prostate cancer, cholangiocarcinoma, thyroid carcinoma, lung cancer, hepatocellular carcinoma and breast cancer, and suggested an involvement between ENO1 and tumor progression [45–51]. However, Lomnytska [52] found that the expression level of ENO1 was markedly reduced, and was associated with favorable prognosis in EC, suggesting ENO1 functions as a tumor suppressor in EC. In contrast, we observed that mRNA and protein levels of ENO1 were robustly upregulated in EC and that elevated expression level of ENO1 was positively correlated with lymph node status. Patients with high ENO1 expression had a markedly shorter overall survival time than patients with low ENO1 expression. These findings were consistent with our functional and mechanistic studies of ENO1, further suggesting it as a potential oncogene during EC pathogenesis.

In conclusion, a high level expression of ENO1 could serve as an unfavorable biomarker for the prognosis of EC. Knocking down ENO1 expression inhibited cell glycolysis, proliferation, migration, invasion, and metastasis via the p85 suppression-mediated inactivation of PI3K/AKT pathway and its downstream signal factors and enhanced the sensitivity of DDP to EC cells *in vitro* and *in vivo*. Our results of the present study suggest that ENO1 acts as an potential therapeutic target in EC.

## MATERIALS AND METHODS

### Cell culture and sample collection

The EC cell lines Ishikawa and HEC-1B were purchased from the Chinese Academy of Sciences (Shanghai, China) and maintained in Dulbecco's modified Eagle's medium (DMEM) (Hyclone, Logan, UT) supplemented with 10% fetal calf serum (ExCell, Shanghai, China). HEEC was purchased from Jiangyin CHIScientific, China and maintained in DMEM and F12 supplemented with 20% fetal calf serum. All cell lines were cultured at 37°C in a humidified atmosphere of 5% CO_2_. 16 fresh NE tissues, 30 fresh primary EC tissues, 25 paraffin-embedded NE specimens and 22 paraffin-embedded endometrial atypical hyperplasia (EAH) and 100 archived paraffin-embedded EC samples were obtained in the Third Affiliated Hospital of Guangzhou Medical School, Guangzhou City, China. Clinical protocols were carried out according to our previous description [27]. For the use of these clinical materials for research purposes, prior consent from the patients and approval from the Ethics Committee of this hospital were obtained. All specimens had confirmed pathological diagnosis and were staged according to FIGO 2009.

### RNA Isolation, reverse transcription, and qRT-PCR

RNA was extracted from the Ishikawa and HEC-1B cell lines, EC tissues and NE tissues using Trizol (Takara, Shiga, Japan). For ENO1, RNA was transcribed into cDNA and amplified with specific sense primers. The primer sequences were the same as our previous report in glioma [27]. For LDHA, the primer sequence was LDHA-sense: AAACGCGCCTTAATTTAGTCCA, LDHA-antisense: CAGCCGCTTCCAATAATACGG. The assays were performed in accordance with manufacturer's instructions (Takara, Shiga, Japan). PCR reactions for each gene were repeated three times. ARF5 was used as internal control.

### Western blotting analysis

Western blotting was carried out according as described [27] with rabbit polyclonal anti-ENO1 and LDHA antibodies (1:1000; Proteintech, USA), anti-Rb, pRb (Ser780), PI3K, pPI3K (Tyr458), AKT, pAKT (Ser473), E2F1, p27, c-Myc, Snail, E-Cadherin and N-Cadherin antibodies (1:1000; Cell Signaling Technology, Danvers, USA). An HRP-conjugated anti-rabbit IgG antibody was used as the secondary antibody (Zhongshan, Beijing, China). Enhanced chemiluminescence reagents were used to detect the signals (Pierce, Rockford, IL).

### Establishment of EC cell lines with stable expression of eno1 short hairpin RNA

The preparation of lentiviruses expressing human ENO1 short hairpinRNA (shRNA-9449, 9450, 9452) (Table 4) were performed by Shanghai Genechem (Genechem, Shanghai, China), performed using the pLVTHM-GFP lentiviral RNAi expression system. The full-length ENO1-RFP (ENO1), RFP empty vector (PLV-Ctr) lentiviruses were performed by Shanghai Genechem (Genechem, Shanghai, China). Ishikawa, HEC-1B and HEEC cells were infected with lentiviral particles containing specific or negative control vectors, and polyclonal cells with GFP or RFP signals were selected for further experiments using FACS flow cytometry.

**Table 4:** shRNA sequences for ENO1

shENO1		Sequence
A	Sense	5′-CCGGAATGTCATCAAGGAGAAATATCTCGAGATATTTCTCCTTGATGACATTTTTTTG-3′
	Antisense	5′-AATTCAAAAAAATGTCATCAAGGAGAAATATCTCGAGATATTTCTCCTTGATGACATT-3′
B	Sense	5′-CCGGCGTGAACGAGAAGTCCTGCAACTCGAGTTGCAGGACTTCTCGTTCACGTTTTG-3′
	Antisense	5′-AATTCAAAACGTGAACGAGAAGTCCTGCAACTCGAGTTGCAGGACTTCTCGTTCACG-3′
C	Sense	5′-CCGGCCACTGTTGAGGTTGATCTCTCTCGAGAGAGATCAACCTCAACAGTGGTTTTTG-3′
	Antisense	5′-AATTCAAAAACCACTGTTGAGGTTGATCTCTCTCGAGAGAGATCAACCTCAACAGTGG-3′

### Transient transfection with siRNAs and p85α plasmid or PI3K inhibitor Ly294002

Small-interfering RNA (siRNA) for ENO1 was designed and synthesized by Guangzhou RiboBio (RiboBio Inc, China). p85α plasmid was purchased in Biosense technologies, guangzhou, China. Three siRNAs targeting on ENO1 gene were designed and synthesized, the most effective siRNA (siENO1) identified by Real Time-PCR was applied for the further experiments. The sequence of siENO1 is: sense: 5′-GCAUUGGAGCAGAGGUUUAdTdT-3′-203′-anti-sense: 3′-dTdTCGUAACCUCGUCUCCAAAU-5′-. Twenty-four hours prior to transfection, EC cells Ishikawa, HEC-1B and HEEC were plated onto a 6-well plate or a 96-well plate (Nest Biotech, China) at 30–50% confluence. Constructs were then transfected into cells using TurboFect TM siRNA Transfection Reagent (Fermentas, Vilnius, Lithuania) according to the manufacturer's protocol. Cells were collected after 48–72 h for further experiments. PI3K inhibitor Ly294002 was bought from Sigma. 1.5 × 10^4^ cells were seeded in 6-well plates. After culturing for 24 h, cells were treated with or without LY294002 at the 10 μM concentration, cells were collected after 48 h for further experiments.

### MTT assay

3-(4, 5-dimethylthiazol-2-yl)-2, 5-diphenyltetrazolium bromide (MTT) assay was used to evaluate the rate of *in vitro* cell proliferation. Cells were seeded in 96-well plates at a density of 1000 cells/well. For shRNA-ENO1 and overexpressed ENO1, cells were incubated for 7 days. For siRNA-ENO1, cells were incubated for 3 days. Twenty microliters of MTT (5 mg/ml) (Sigma, St. Louis, MO) was added to each well and incubated for 4 h. At the end of incubation, supernatants were removed, and 150μl of DMSO (dimethyl sulfoxide) (Sigma, St. Louis, MO) was added to each well. The absorbance value (OD) of each well was measured at 490 nm. For each experimental condition, eight wells were used. Experiments were performed thrice.

### Cell cycle analysis

Cell cycle examination was carried out according to a previous description [39]. The DNA content of labeled cells was acquired using FACS cytometry assay (BD Biosciences).

### Colony formation assay

Cells were plated in 6-well culture plates at 100 cells/well. After incubation for 15 days at 37°C, cells were washed twice with PBS and stained with Giemsa solution. The number of colonies containing < 50 cells was counted under a microscope. The colony formation efficiency was calculated as (number of colonies/number of cells inoculated) × 100%.

### Immunofluorescence

EC cells were seeded on coverslips in 6-well plate and cultured overnight. Subsequently cells were fixed in 3.5% paraformaldehyde, permeabilized in KB solution and 0.2% Triton X-100 at room temperature. After the blocking solution was washed out, cells were incubated with a primary antibody (ENO1) (diluted in KB) 30–45 min at 37°C and subsequently washing with KB twice. After incubating 30–45 min at 37°C with secondary antibody (diluted in KB), washing with KB again, the coverslips were then mounted onto slides with mounting solution containing 0.2 mg/ml DAPI and sealed with nail polish. The F-actin status was analyzed by phalloidin staining (Abbkine, Inc., Redlands, CA, USA) for 45 min at room temperature. Slides were stored at 4°C in a dark box and observed under a fluorescent microscope.

### *In vivo* tumorigenesis in nude mice

A total of 1 × 10^6^ logarithmically grown Ishikawa cells transfected with GFP-shENO1 or control PLVTHM-GFP vector in 0.1 ml DMEM medium were subcutaneously injected into the left flank of 4–6-week-old male BALB/c nu/nu mice. Mice were maintained in a barrier facility on HEPA-filtered racks. The animals were fed an autoclaved laboratory rodent diet. All animal studies were conducted in accordance with the principles and procedures outlined in Southern Medical University Guide for the Care and Use of Animals under assurance number SCXK (Guangdong) 2008–0002. After 19 days the mice were killed and tumor tissues were excised and weighed.

### Cell migration and invasion assays

For cell migration assays, 1 × 10^5^ cells in 100μl DMEM medium without FCS were seeded on a fibronectincoated polycarbonate membrane insert in a transwell apparatus (Costar, MA). In the lower chamber, 600μl DMEM with 10% FCS was added as chemoattractant. After the cells were incubated for 16 h at 37°C in a 5% CO_2_ atmosphere, the insert was washed with PBS, and cells on the top surface of the insert were removed with a cotton swab. Cells adhering to the lower surface were fixed with methanol, stained with Giemsa solution and counted under a microscope in five predetermined fields (200 ×). All assays were independently repeated three times. For the cell invasion assay, the procedure was similar to the cell migration assay, except that the transwell membranes were precoated with 24 μg/μl Matrigel (R&D Systems, USA).

### *In vivo* metastasis assays

To evaluate the *in vivo* liver metastatic potential of cancer cells, 5 × 10^6^ Ishikawa-shENO1 and Ishikawa-PLVTHM cells were injected into nude mice (*n* = 5 for each group) through the spleen, respectively. Whole-body optical images were visualized to monitor primary tumor growth and formation of metastatic lesions. Two months later, all mice were killed, individual organs were removed.

### MTT cytotoxicity assay

Cis-diamminedichloroplatinum (Cisplatin, DDP) (Qilu Pharmo Co. Ltd, China) was resuspended in PBS (0.5mg/ml) and stored at −20°C. Drug sensitivity test was determined by MTT assay. Cells were seeded in 96-well plates in 100 μl DMEM medium supplemented with 10% FBS at 5 × 10^3^ cells/well. Once the cells attached, they were treated with 2.5, 5, 10, 20 or 40 μM Cisplatin (0.5mg/ml) and incubated at 37°C in 5% CO_2_ for 48 h. Subsequently, 10 μl of MTT (5 mg/ml) (Sigma, StLouis, MO, USA) was added to each well, and the plates were incubated at 37°C for 4 h. At the end of incubation, the supernatants were removed and 100 μl of DMSO (Sigma) was added to each well. The absorbance value (OD) of each well was measured at 490 nm. The calculated rates were then used for curve fitting and half maximal inhibitory concentration (IC50) calculations. Experiments were performed three times.

### Treatment experiment on nude mice

*In vivo* experiment was approved by the Animal Care and Use Committee of Southern Medical University. All mice of 4–5 weeks old and 16–20 g in weight were provided by the Central Animal Facility of Southern Medical University. To establish an EC mouse model, 6 × 10^5^ sh-ENO1 Ishikawa cells and their controls in 0.2 mL buffered saline were subcutaneously injected into the back of nude mice (BALB/C, nu/nu, 4–6 weeks) and tumors were allowed to grow for 10 days. The tumor volume was measured with a caliper every three days and calculated by the formula: Volume = 1/2 × length × width^2^, where length represented the longest tumor diameter and width represented the shortest tumor diameter. When tumor volume reached 30–100 mm^3^, the animals were randomized into three groups for therapy testing. Mice treated with sh-ENO1-Ishikawa cells and PLVTHM-Ishikawa cells were intratumorally injected with normal saline and cisplatin, respectively. Mice treated with PLVTHM-Ishikawa cells were only intratumorally injected with cisplatin. All mice were sacrificed after 18 days and the tumors were carefully dissected.

### Immunohistochemical staining

The paraffin sections prepared from *in vivo* experiments were applied to immunohistochemistry assays for detecting protein expression levels of ENO1, PI3K (p85) and c-Myc proteins. The indirect streptavidin-peroxidase method was used as the manufacturer's introduction. The immunohistochemicallystained tissue sections were reviewed separately by two pathologists. The antibodies were rabbit anti-ENO1 antibody (1:50, Proteintech, USA) and anti-PI3K (P85) antibody (1:20, Proteintech, USA) and anti-c-Myc antibody (1:50, Proteintech, USA), respectively.

### Metabolic profiling

Metabolomic profiles were obtained to assess the relative distribution of various cellular metabolites of immortalized HEEC and EC cells. Cells were collected and quickly frozen. Further sample preparation, metabolic profiling, peak identification and curation were performed by Metabolon (Durham, NC, USA) using their described methods [53].

### Immunohistochemistry and evaluation of staining

Immunohistochemistry and evaluation of staining of ENO1 (1 : 50, Proteintech, USA) were performed in EC and NE tissues according to a previous description [54].

### Statistical analysis

SPSS 13.0 (SPSS Inc, Chicago, IL, USA) and Graph Pad Prism 5.0 software (GraphPad Software, Inc., La Jolla, CA, USA) were used to analyze all data for statistical significance. The chi-squared test was applied to examine the relationship between ENO1 expression levels and clinicopathological characteristics. Survival analysis was performed using Kaplan-Meier method. Multivariate Cox proportional hazards method was used for analyzing the relationship between variables and patient survival time. Statistical signifiance was denoted as follows: *p < 0.05, **p < 0.01, ***p < 0.001. Data shown is mean ± SEM unless indicated otherwise.

## SUPPLEMENTARY FIGURES



## References

[1] Siegel R, Naishadham D, Jemal A (2013). Cancer statistics. CA Cancer J Clin.

[2] Azueta A, Gatius S, Matias-Guiu X (2010). Endometrioid carcinoma of the endometrium: pathologic and molecular features. Semin Diagn Pathol.

[3] Wong YF, Cheung TH, Lo KW, Yim SF, Siu NS, Chan SC, Ho TW, Wong KW, Yu MY, Wang VW, Li C, Gardner GJ, Bonome T (2007). Identification of molecular markers and signaling pathway in endometrial cancer in Hong Kong Chinese women by genome-wide gene expression profiling. Oncogene.

[4] Zhang Y, Liu Z, Yu X, Zhang X, Lü S, Chen X, Lü B (2010). The association between metabolic abnormality and endometrial cancer: a large case-control study in China. Gynecol Oncol.

[5] Dedes KJ, Wetterskog D, Ashworth A, Kaye SB, Reis-Filho JS (2011). Emerging therapeutic targets in endometrial cancer. Nat Rev Clin Oncol.

[6] Vander Heiden MG, Cantley LC, Thompson CB (2009). Understanding the Warburg effect: the meta-bolic requirements of cell proliferation. Science.

[7] Merkulova T, Dehaupas M, Nevers MC, Creminon C, Alameddine H, Keller A, Merkulova T, Dehaupas M, Nevers MC, Creminon C, Alameddine H, Keller A (2000). Differential modulation of alpha, beta and gamma enolase isoforms in regenerating mouse skeletal muscle. Eur J Biochem.

[8] Ghosh AK, Steele R, Ray RB (2006). Knockdown of MBP-1 in human prostate cancer cells delays cell cycle progression. J Biol Chem.

[9] Cairns RA, Harris IS, Mak TW (2011). Regulation of cancer cell metabolism. Nat Rev Cancer.

[10] Altenberg B, Greulich KO (2004). Genes of glycolysis are ubiquitously overexpressed in 24 cancer classes. Genomics.

[11] He P, Naka T, Serada S, Fujimoto M, Tanaka T, Hashimoto S, Shima Y, Yamadori T, Suzuki H, Hirashima T, Matsui K, Shiono H, Okumura M (2007). Proteomics-based identification of α-enolase as a tumor antigen in non-small lung cancer. Cancer Sci.

[12] Ralhan R, Masui O, Desouza LV, Matta A, Macha M, Siu KW (2011). Identification of proteins secreted by head and neck cancer cell lines using LC-MS/MS: strategy for discovery of candidate serological biomarkers. Proteomics.

[13] Seweryn E, Pietkiewicz J, Bednarz-Misa IS, Ceremuga I, Saczko J, Kulbacka J, Gamian A (2009). Localization of enolase in the subfractions of a breast cancer cell line. Z Naturforsch C.

[14] Yu Z, Willmarth NE, Zhou J, Katiyar S, Wang M, Liu Y, McCue PA, Quong AA, Lisanti MP, Pestell RG (2010). microRNA 17/20 inhibits cellular invasion and tumor metastasis in breast cancer by heterotypic signaling. Proc Natl Acad Sci USA.

[15] Liang J, Slingerland JM (2003). Multiple roles of the PI3K/PKB (Akt) pathway in cell cycle progression. Cell Cycle.

[16] Shin HJ, Park YH, Kim SU, Moon HB, Park do S, Han YH, Lee CH, Lee DS, Song IS, Lee DH, Kim M, Kim NS, Kim DG (2011). Hepatitis B virus X protein regulates hepatic glucose homeostasis via activation of inducible nitric oxide synthase. J Biol Chem.

[17] Diamond DL, Syder AJ, Jacobs JM, Sorensen CM, Walters KA, Proll SC, McDermott JE, Gritsenko MA, Zhang Q, Zhao R, Metz TO, Camp DG, Waters KM (2010). Temporal proteome and lipidome profiles reveal hepatitis C virus-associated reprogramming of hepatocellular metabolism and bioenergetics. PLoS Pathog.

[18] Delgado T, Carroll PA, Punjabi AS, Margineantu D, Hockenbery DM, Lagunoff M (2010). Induction of the Warburg effect by Kaposi's sarcoma herpesvirus is required for the maintenance of latently infected endothelial cells. Proc Natl Acad Sci USA.

[19] Bhatt AP, Jacobs SR, Freemerman AJ, Makowski L, Rathmell JC, Dittmer DP, Damania B (2012). Dysregulation of fatty acid synthesis and glycolysis in non-Hodgkin lymphoma. N Proc Natl Acad Sci USA.

[20] Vastag L, Koyuncu E, Grady SL, Shenk TE, Rabinowitz JD (2011). Divergent effects of human cytomegalovirus and herpes simplex virus-1 on cellular metabolism. PloSPathog.

[21] Pancholi V (2001). Multifunctional alpha-enolase: its role in diseases. Cell Mol Life Sci.

[22] Díaz-Ramos À, Roig-Borrellas A, García-Melero A, López-Alemany R (2012). α-Enolase, amultifunctional protein: its role on pathophysiological situations. J Biomed Biotechnol.

[23] Hamaguchi T, Iizuka N, Tsunedomi R, Hamamoto Y, Miyamoto T, Iida M, Tokuhisa Y, Sakamoto K, Takashima M, Tamesa T, Oka M (2008). Glycolysis moduleactivated by hypoxia-inducible factor 1alpha is related to the aggressive phenotype of hepatocellular carcinoma. Int J Oncol.

[24] Wu W, Tang X, Hu W, Lotan R, Hong WK, Mao L (2002). Identiﬁcation and validation of metastasis-associated proteins in head and neck cancer cell lines by two-dimensional electrophoresis and mass spectrometry. Clin Exp Metastasis.

[25] Yoshida A, Okamoto N, Tozawa-Ono A, Koizumi H, Kiguchi K, Ishizuka B, Kumai T, Suzuki N (2013). Proteomic analysis of differential protein expression by brain metastases of gynecological malignancies. Human Cell.

[26] Chang YS, Wu W, Walsh G, Hong WK, Mao L (2003). Enolase-alpha is frequently down-regulated in non-small cell lung cancer and predicts aggressive biological behavior. Clin Cancer Res.

[27] Song Y, Luo Q, Long H, Hu Z, Que T, Zhang X, Li Z, Wang G, Yi L, Liu Z, Fang W, Qi S (2014). Alpha-enolase as a potential cancer prognostic marker promotes cell growth, migration, and invasion in glioma. Mol Cancer.

[28] Shim H, Dolde C, Lewis BC, Wu CS, Dang G, Jungmann RA, Dalla-Favera R, Dang CV (1997). c-Myc transactivation of LDH-A: implications for tumor metabolism and growth. Proc Natl Acad Sci U S A.

[29] Yecies JL, Manning BD (2011). Transcriptional control of cellular metabolism by mTOR signaling. Cancer Res.

[30] Wallace DC (2005). Mitochondria and cancer: Warburg addressed. Cold Spring Harb Symp Quant Biol.

[31] Xiao L, Hu ZY, Dong X, Tan Z, Li W, Tang M, Chen L, Yang L, Tao Y, Jiang Y, Li J, Yi B (2014). Targeting Epstein-Barr virus oncoprotein LMP1-mediated glycolysis sensitizes nasopharyngeal carcinoma to radiation therapy. Oncogene.

[32] Chau BN, Wang JY (2003). Coordinated regulation of life and death by RB. Nat Rev Cancer.

[33] Yang W, Shen J, Wu M, Arsura M, FitzGerald M, Suldan Z, Kim DW, Hofmann CS, Pianetti S, Romieu-Mourez R, Freedman LP, Sonenshein GE (2001). Repression of transcription of the p27(Kip1) cyclin dependent kinase inhibitor gene by c-Myc. Oncogene.

[34] Gartel AL, Shchors K (2003). Mechanisms of c-myc-mediated transcriptional repression of growth arrest genes. Cell.

[35] Wang C, Hou X, Mohapatra S, Ma Y, Cress WD, Pledger WJ (2005). Activation of p27Kip1 expression by E2F1. A negative feedback mechanism. J Biol Chem.

[36] Schmidt EV (2004). The role of c-myc in regulation of translation initiation. Oncogene.

[37] Zhou BP, Deng J, Xia W, Xu J, Li YM, Gunduz M, Hung MC (2004). Dual regulation of Snail by GSK-3beta-mediated phosphorylation in control of epithelial-mesenchymal transition. Nat. Cell Biol.

[38] Kume K, Haraguchi M, Hijioka H, Ishida T, Miyawaki A, Nakamura N, Ozawa M (2013). The transcription factor Snail enhanced the degradation of E-cadherin and desmoglein 2 in oral squamous cell carcinoma cells. Biochem Biophys Res Commun.

[39] Liu Z, Mai C, Yang H, Zhen Y, Yu X, Hua S, Wu Q, Jiang Q, Zhang Y, Song X, Fang W (2014). Candidate tumour suppressor CCDC19 regulates miR-184 direct targeting of C-Myc thereby suppressing cell growth in non-small cell lung cancers. J Cell Mol.

[40] Bader AG, Kang S, Zhao L, Vogt PK (2005). Oncogenic PI3K deregulates transcription and translation. Nat Rev Cancer.

[41] Julien S, Puig I, Caretti E, Bonaventure J, Nelles L, van Roy F, Dargemont C, de Herreros AG, Bellacosa A, Larue L (2007). Activation of NF-kappaB by Akt upregulates snail expression and induces epithelium mesenchyme transition. Oncogene.

[42] Wang H, Wang HS, Zhou BH, Li CL, Zhang F, Wang XF, Zhang G, Bu XZ, Cai SH, Du J (2013). Epithelial-mesenchymal transition (EMT) induced by TNF-alpha requires AKT/GSK-3beta-mediated stabilization of snail in colorectal cancer. PLoS ONE.

[43] Shen XY, Xiang Y (2010). Endometrial cancer chemotherapy. International Journal of Obstetrics and Gynecology.

[44] Moxley KM, McMeekin DS (2010). Endometrial carcinoma: a review of chemotherapy, drug resistance, and the search for new agents. Oncologist.

[45] Cappello P, Rolla S, Chiarle R, Principe M, Cavallo F, Perconti G, Feo S, Giovarelli M, Novelli F (2013). Vaccination with ENO1 DNA prolongs survival of genetically engineered mice with pancreatic cancer. Gastroenterology.

[46] Yu L, Shi J, Cheng S, Zhu Y, Zhao X, Yang K, Du X, Klocker H, Yang X, Zhang J (2012). Estrogen promotes prostate cancer cell migration via paracrine release of ENO1 from stromal cells. Mol Endocrinol.

[47] Yonglitthipagon P, Pairojkul C, Bhudhisawasdi V, Mulvenna J, Loukas A, Sripa B (2012). Proteomics-based identification of alpha-enolase as a potential prognostic marker in cholangio carcinoma. Clin Biochem.

[48] Chu PY, Hsu NC, Liao AT, Shih NY, Hou MF, Liu CH (2011). Overexpression of alpha-enolase correlates with poor survival in canine mammary carcinoma. BMC Vet Res.

[49] Trojanowicz B, Sekulla C, Lorenz K, Kohrle J, Finke R, Dralle H, HoangVu C (2010). Proteomic approach reveals novel targets for retinoic acid-mediated therapy of thyroid carcinoma. Mol Cell Endocrinol.

[50] Tsai ST, Chien IH, Shen WH, Kuo YZ, Jin YT, Wong TY, Hsiao JR, Wang HP, Shih NY, Wu LW (2010). ENO1, a potential prognostic head and neck cancer marker, promotes transformation partly via chemokine CCL20 induction. Eur J Cancer.

[51] Ho JA, Chang HC, Shih NY, Wu LC, Chang YF, Chen CC, Chou C (2010). Diagnostic detection of human lung cancer-associated antigen using a gold nanoparticle-based electrochemical immunosensor. Anal Chem.

[52] Lomnytska MI, Becker S, Gemoll T, Lundgren C (2012). Impact of genomic stability on protein expression in endometrioid endometrial cancer. Br J Cancer.

[53] Reitman ZJ, Jin G, Karoly ED, Spasojevic I, Yang J, Kinzler KW, He Y, Bigner DD, Vogelstein B, Yan H (2011). Profiling the effects of isocitrate dehydrogenase 1 and 2 mutations on the cellular metabolome. Proc Natl Acad Sci USA.

[54] Wang L, Jiang Q, Hua S, Zhao M, Wu Q, Fu Q, Fang W, Guo S (2012). High nuclear expression of HDGF correlates with disease progression and poor prognosis in human endometrial carcinoma. Dis Markers.

