# Supplementary material for: Enolase-1 is a therapeutic target in endometrial carcinoma
Source: Oncotarget. 2015 Apr 24;6(17):15610–27. doi: 10.18632/oncotarget.3639 (PMC4558174; doi:10.18632/oncotarget.3639)
Supplement: Supplementary file 1 [file oncotarget-06-15610-s001.pdf]

SUPPLEMENTARY FIGURES

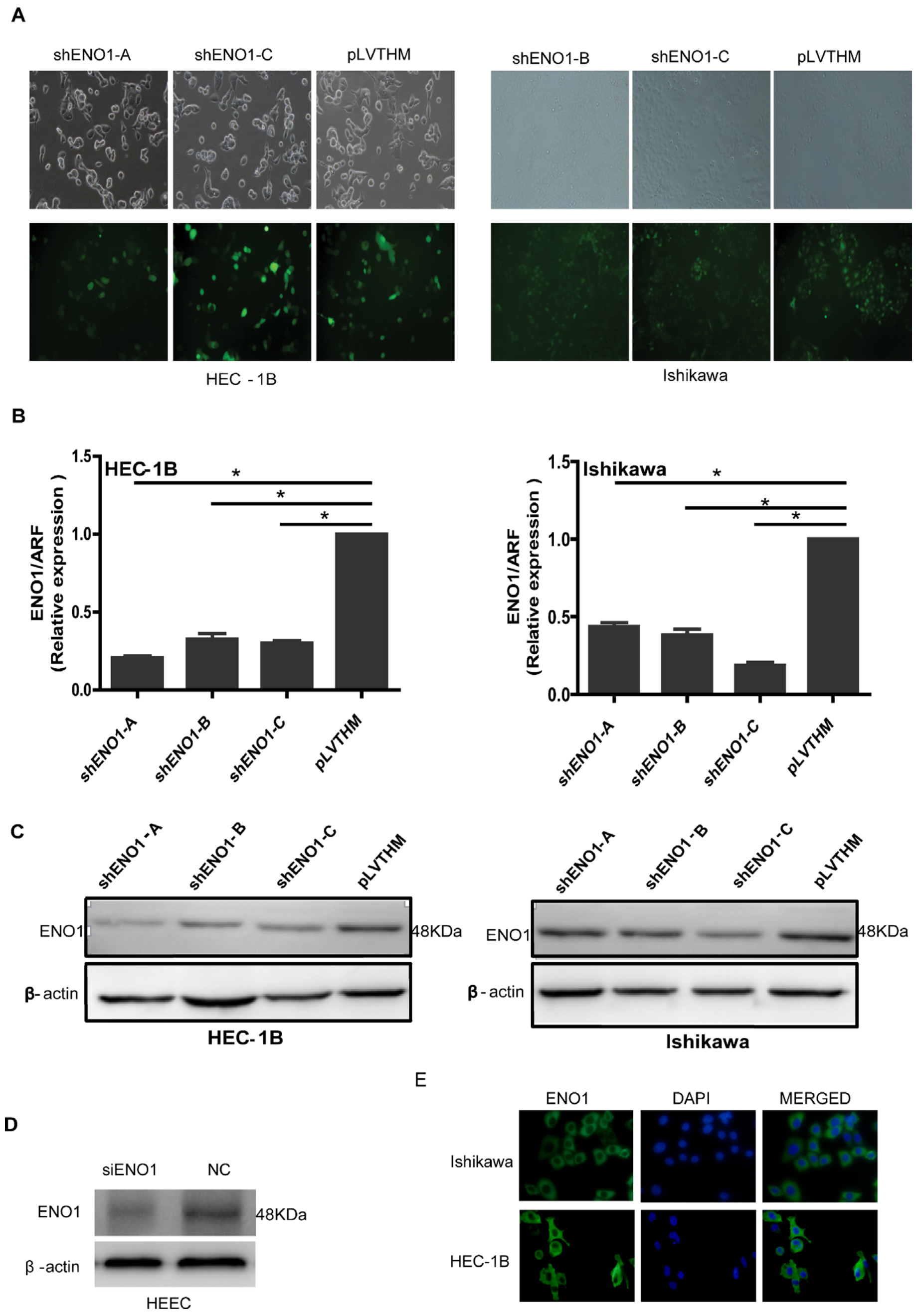

Supplementary Figure S1: Efficiency of shRNA or siRNA knock down of ENO1 in human EC cell lines HEC-1B and Ishikwawa and HEEC. A. HEC-1B and Ishikawa were transfected by different lentivirus which contain sh-ENO1 and PLVTHM. **B.** RT-PCR showing transcriptional levels of the ENO1 gene with ARF used as a loading control. Bar graph showed the relative expression of mRNA among the groups. Data were presented as mean ± SEM for three independent experiments (*$P < 0.05$). **C.** Western blotting showing protein expression levels in shENO1 or PLVTHM cells. A representative image of three different experiments was shown. β-actin served as a loading control. **D.** Western blotting showing protein expression levels in siENO1-HEEC and HEEC-NC cells. Each experiment was repeated three times. **E.** Immunofluorescence staining of ENO1 in Ishikawa and HEC-1B cells (magnification, × 1000).

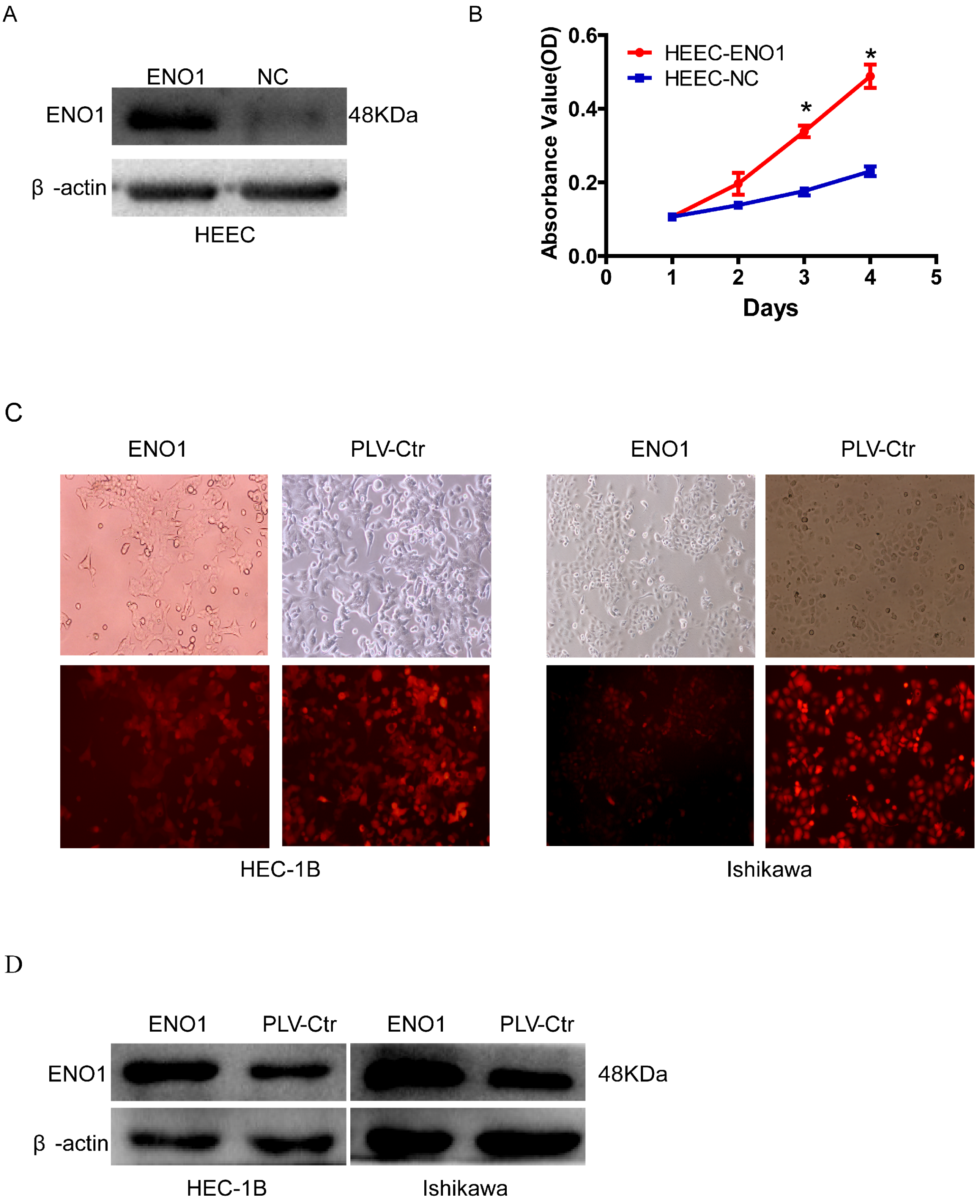

Supplementary Figure S2: Overexpressing ENO1 in HEEC and EC cells. A. Overexpressing ENO1 increased the protein level of ENO1 in HECC detected by western blotting. β-actin served as the internal control. Each experiment was repeated three times. **B.** MTT assay showed that elevated ENO1 induced cell proliferation in HEEC. Data were presented as mean ± SEM for three independent experiments (**P* < 0.05). **C.** HEC-1B and Ishikawa were transfected by lentivirus which contain full-length ENO1 and RFP empty vector (PLV-Ctr). **D.** Western blotting showing protein expression levels in ENO1 or PLV-Ctr cells. A representative image of three different experiments was shown. β-actin served as a loading control.

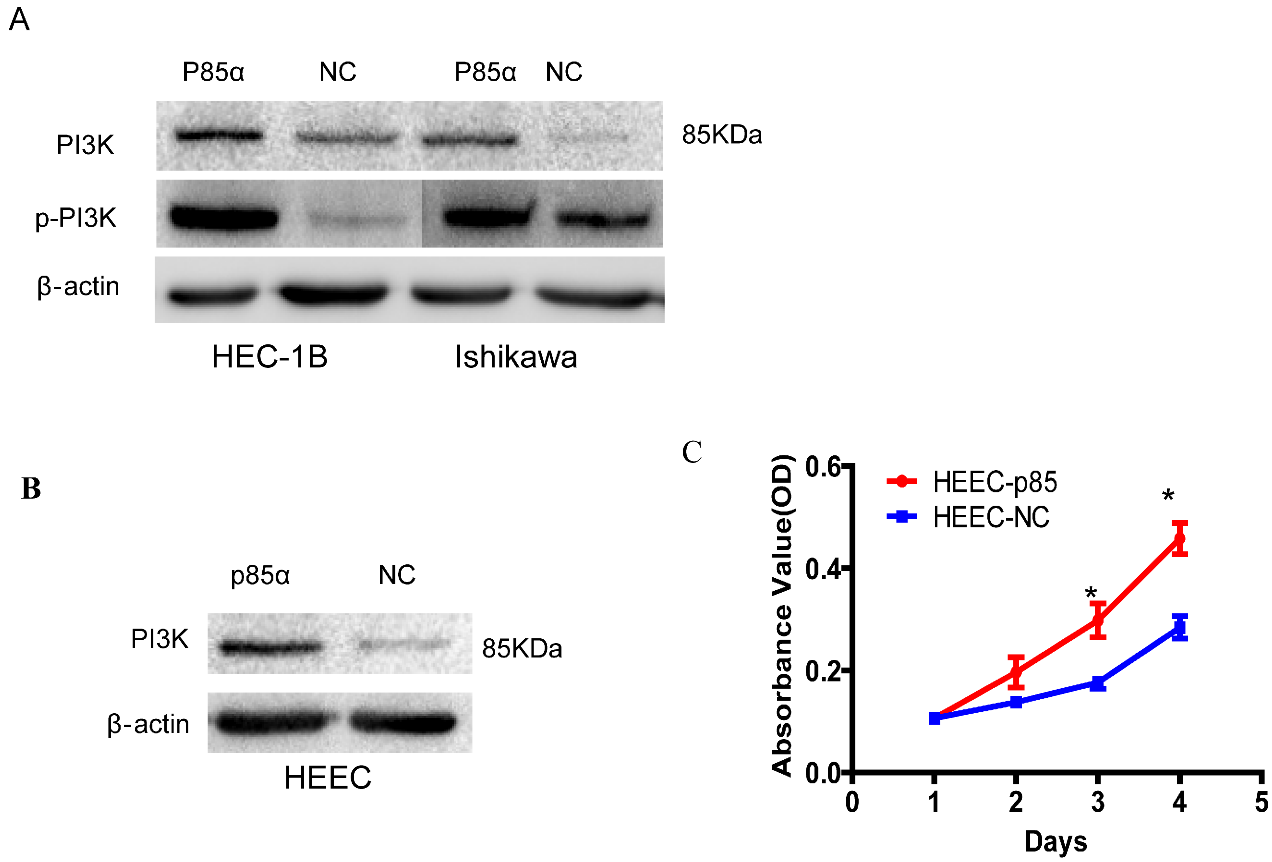

Supplementary Figure S3: Overexpression of P85 expression in EC cells and HEEC. A. Overexpressing p85 increased the protein level of PI3K and p-PI3K in EC cells detected by western blotting. β-actin served as the internal control. Each experiment was repeated three times. **B.** Western blotting showing PI3K protein expression levels in P85 overexpressed HEEC and its control cells. A representative image of three different experiments was shown. β-actin served as a loading control. **D.** Effect of p85 overexpressed on HEEC cell proliferation as measured by MTT assay. Data were presented as mean ± SEM for three independent experiments (*$P < 0.05$).
